# Acid selenites as new selenium precursor for CdSe quantum dot synthesis

**DOI:** 10.1016/j.heliyon.2023.e23837

**Published:** 2023-12-15

**Authors:** João B. Souza Junior, Beatriz Mouriño, Marcelo H. Gehlen, Daniel A. Moraes, Jefferson Bettini, Laudemir C. Varanda

**Affiliations:** a Colloidal Materials Group, Physical-Chemistry Department, Instituto de Química de São Carlos, Universidade de São Paulo, 13566-590, São Carlos - SP, Brazil; b Brazilian Nanotechnology National Laboratory (LNNano), Brazilian Center for Research in Energy and Materials (CNPEM), 13083-970, Campinas - SP, Brazil

**Keywords:** 00-01, 99-00, Acid selenites, CdSe, Quantum dots, Colloidal synthesis

## Abstract

Chemical precursors for nanomaterials synthesis have become essential to tune particle size, composition, morphology, and unique properties. New inexpensive precursors investigation that precisely controls these characteristics is highly relevant. We studied new Se precursors, the acid selenites (R–O–SeOOH), to synthesize CdSe quantum dots (QDs). They were produced at room temperature by the  reaction with alcohols having different alkyl chains and were characterized by ^1^H NMR confirming their structures. This unprecedented precursor generates high-quality CdSe nanocrystals with narrow size distribution in the zinc-blend structure showing controlled optical properties. Advanced characterization detailed the CdSe structure showing stacking fault defects and its dependence on the used R–O–SeOOH. The QDs formation was examined using a time-dependent growth kinetics model. Differences in the nanoparticle surface structure influenced the optical properties, and they were correlated to the Se-precursor nature. Small alkyl chain acid selenites generally lead to more controlled QDs morphology, while the bigger alkyl chain leads to slightly upper quantum yields. Acid selenites can potentially replace Se-precursors at competitive costs in the metallic chalcogenide nanoparticles.  is chemically stable, and alcohols are cheap and less toxic than the reactants used today, making acid selenites a more sustainable Se precursor.

## Highlights

• Acid selenites (R-O-SeOOH) from  and alcohols of different alkyl chain reactions at room temperature are described as new Se precursors.
• This unprecedented precursor generates high-quality CdSe nanocrystals with narrow size distribution and controlled optical properties.
• Acid selenites can potentially replace Se-precursors in metallic chalcogenide and other Se-based materials at competitive costs.
• Acid selenites are less toxic and a more sustainable Se precursor.

Acid selenites (R-O-SeOOH) from  and alcohols of different alkyl chain reactions at room temperature are described as new Se precursors.

## Graphical abstract

**Figure:**
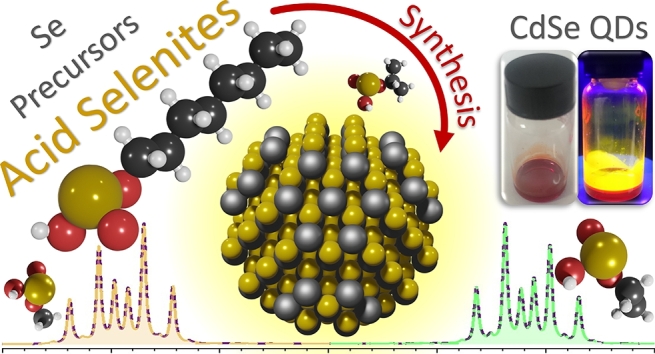

## 1 Introduction

Chemically-prepared colloidal nanocrystals (NCs) became a high-demand nanomaterial due to their potential technological applications [1]. Spherical semiconductor NCs with diameters in the quantum confinement regime (below the Bohr radius), so-called quantum dots (QDs), are intensively pursued because of their optical properties [2], [3], [4]. The ability to tune the luminescence of QDs with extreme color purity (narrow emission) throughout the visible spectrum raises the significance of using these nanomaterials in optoelectronic devices [5], [6], [7]. Moreover, QDs have also been studied for several technologies, including optimized memory techniques [8], neuromorphic computing systems [8], quantum information [9], light-emitting diodes (LEDs) [10], solar cells [11], transistors [12], lasers [13], photocatalytic hydrogen (H_2_) production [14], and nanomedicine [15]. Colloidal QDs can be used in solution-processed methods for device fabrication, which can be designed as a low-cost process compared to traditional industry methods [6]. Nowadays, QDs have already been in the market in products such as high-definition displays (QLEDS TVs) and pure color LEDs. Thus, a synthetic protocol that leads to narrow size distribution must be used to achieve their full luminescence capabilities. Therefore, wet chemical solution synthesis of QD using lower-cost reactants that lead to monodisperse NCs can facilitate future technologies [16].

II-VI semiconductor QDs have shown the highest advances in synthesis methods, mainly due to their chemical stability and reaction pathways possibilities compared to III-V QDs [17]. Among II-VI semiconductors, CdSe QDs have roused the interest of researchers for years, in part due to the possibility of tuning its bang gap, increasing the band gap as the QD size decreases, controlling its luminescence with narrow emission profile for almost all visible spectrum [18], [19]. Typically, CdSe QDs are produced by wet chemical synthesis using toxic and expensive phosphorous-based compounds as surfactants. Cadmium precursor varies from C–Cd organocadmium compounds (like dimethyl cadmium), (O)C–O–Cd carboxylates (acetate, oleate, and myristate), (O)(OH)P–O–Cd phosphonic acid (octadecyl phosphonic acid, ODPA; hexyl phosphonic acid, HPA; tetradecyl phosphonic acid, TDPA), among others [20]. However, selenium precursors are primarily based on TOP-Se chemistry, which is synthesized by the reaction of elemental Se with trioctylphosphine (TOP) at mild temperatures or by using bis(trimethylsilyl) selenium precursor [18], [19]. Both selenium precursors are highly unstable, presenting volatility, air-sensitivity, and pyrophoric compounds formation with acute toxicity, making the synthesis procedure costly and less environmentally friendly [21], [22]. However, high-quality CdSe QDs are obtained using these selenium precursors, and the knowledge regarding these precursors is extensively explored in the literature. Chen et al. introduced selenium dioxide (), a known oxidizing agent in organic chemistry, as a precursor for CdSe QDs synthesis [23].  is a stable white solid at room temperature with less toxicity than TOP-Se. To obtain CdSe,  needs to be reduced from  to . In the presence of 1-octadecene (ODE), the  can be reduced by oxidizing the vinyl group of ODE or mediating the dehydrogenation of the hydrocarbon chains [23]. Therefore, exploring the chemistry of selenium is another opportunity to create new pathways to produce CdSe QDs.

The classical nucleation and growth theory of homogeneous nanoparticle synthesis state that burst nucleation must occur at the initial solid formation [24]. This burst nucleation populates the initial growth stage with homogeneous clusters of reasonable sizes, which can grow, producing narrower size distribution nanoparticles [24], [25]. Thus, a supersaturated solution of a highly reactive compound is generally required to fulfill the burst nucleation [24], [25]. The CdSe monomer unit that generates solid CdSe QDs is obtained from  species. This species occurs due to intermediate reduction reactions of the stable  compound () prior to the formation of the monomer in the solution. Chen et al. showed that the kinetics of nanoparticle formation using  directly in the reaction medium is relatively slow. According to the authors, the first nuclei were observed after 30 s, and the final reaction took about 60 min [23]. Bullen et al. reported faster growth kinetics for CdSe QDs in ODE using TOP-Se precursor, starting and ending around 5 s to 200 s [26]. However, the chemical synthesis features are related to other factors such as precursors, solvent, concentration and nature of the ligands, and the reaction temperature. Thus, a direct comparison of nanoparticle growth kinetics results is complex. Nevertheless, a simple statement can be established that  leads to a slower reaction than TOP-Se, which does not necessarily compromise the CdSe QDs quality. Other TOP-free selenium precursors have been explored, *e.g.*, directly using elemental  in ODE [27], [28] and oleylamine [29], [30],  previously dissolved in ODE or dodecanethiol [22], selenoureas [31], and  [32].

Here we show a fast and straightforward synthesis of a selenium precursor based on acid selenite compounds to produce CdSe QDs. Although the synthesis of acid selenites has been known since the 1930s decade [33], [34], [35], here we report the first use of acid selenites (R–O–SeOOH) as a precursor to producing CdSe QDs. Selenium dioxide powder can be easily dissolved in alcohols (R–OH), leading to highly reactive selenium precursors [33], [34], [35]. Our finds are focused on three alcohols with different hydrocarbons chain lengths, named methanol (), 1-octanol (), and oleyl alcohol (), to demonstrate that their acid selenites products can lead to high-quality CdSe QDs by the hot-injection method. Other alcohols (ethanol, isopropanol, and triethylene glycol) were also evaluated as acid selenite precursors, showing the same effectiveness in preparing controlled-CdSe QDs. The nanoparticle's structure characterization and optical properties are discussed and prove the potential application of acid selenites as a cheap and reliable alternative selenium precursor.

## 2 Experimental section

### 2.1 Chemicals

Cadmium acetylacetonate, (, 99.5%), 1-octadecene (ODE, 90%), oleic acid (OA, 90%), oleylamine (OAm, 70%), selenium dioxide (, 99.9%), 1,2-Hexadecanediol (HDD, 90%), oleyl alcohol (85%), and rhodamine 6G were purchased from Sigma-Aldrich. Methyl alcohol (Met–OH, anhydrous), ethyl alcohol (Et–OH, anhydrous), and 1-octanol (Oct–OH, anhydrous) were purchased from J.T. Baker. All other solvents used were ACS reagent grade and were purchased from Labsynth (Brazil).

### 2.2 Synthesis of R–O–SeOOH precursors

The alkyl acid selenites solutions (2 mol L^−1^) were prepared at room temperature by the reaction of 2 mmol of  in alcohols such as methanol (–, ), 1-octanol (–, ), and oleyl alcohol (–, ). Long-chain alcohols required sonication to help the  powder dissolution and mild heating was used for oleyl alcohol. However, heating was not employed for Met–OH and Oct–OH to avoid solvent evaporation. All R–O–SeOOH precursors formed colorless solutions, except Oleyl–O–SeOOH, which was brownish. R–O–SeOOH precursors were kept in the  atmosphere before injection.

### 2.3 Alkyl acid selenites analysis

^1^H NMR spectra of R–O–SeOOH precursors were obtained using the Agilent Technologies 400/54 Premium Shielded NMR equipment. Chemical shifts are reported in parts per million (ppm) using tetramethylsilane (TMS, ) as a 0 ppm reference. Samples were similarly prepared as described before for R–O–SeOOH precursors. All experiments were performed in deuterated chloroform, .

### 2.4 Synthesis of CdSe quantum dots (QDs)

Syntheses were carried out in a three-neck round-bottom flask under magnetic stirring using a Schlenk line apparatus to control vacuum and  environments. Briefly,  (0.5 mmol), HDD (1 mmol), OA (1 mmol), OAm (1 mmol), and ODE (20 mL) were heated under a vacuum at 100 ^∘^C for 30 min. After that, the atmosphere was replaced by , and the temperature was raised to 220 ^∘^C. Then, the R–O–SeOOH precursor solution (250 μL) was quickly injected into the reaction medium. The QDs size could be controlled by the reaction time by using an ice bath to quench the growth of the nanocrystals or by adding toluene (5 mL) for fast quenching. Growth kinetics were evaluated by withdrawing aliquots (≈ 250 μL) using a syringe, followed by a quick dilution in cold toluene. Purification was carried out using toluene (≈ 10 mL) and isopropanol (≈ 30 mL) in two separated centrifuge tubes, followed by centrifuging (8500 rpm/10 min). The precipitate was re-suspended twice in a toluene-isopropanol mixture, centrifuged, and the supernatant was discarded. The final precipitate was re-suspended in toluene and centrifuged again to remove unreacted precursors, and the final samples were kept in toluene for further characterization. The same procedure was applied to other precursors to demonstrate the methodology versatility: ethanol (–, ), isopropyl alcohol (–, ), and triethylene glycol ().

### 2.5 Structural characterization

X-ray Powder Diffraction (XRD) patterns were obtained using the Bruker D8 Advance diffractometer (Cu *Kα* 1.5418 Å, Ni-filtered). Samples were supported onto Si low background sample holder by directly drop-casting the CdSe toluene suspension. The patterns were recorded from 20^∘^ to 90^∘^ (2*θ*) using a step of 0.02^∘^, 0.5 s/step of exposure time, and accumulating the signal for 4 hours. Transmission electron microscopy (TEM) images were recorded using a corrected TEM Titan Themis Cubed (FEI Company) operating at 300 kV. Samples were prepared by depositing one drop of dilute particle dispersions in toluene onto a carbon-coated copper grid. Average particle size diameter and standard deviation were determined statistically by counting above 200 particles. X-ray photoelectron spectroscopy (XPS) analysis was performed in a K-Alpha X-ray Photoelectron Spectrometer System (Thermo Scientific). The ultra-high vacuum chamber (UHV) operating pressure was $1\times 10^{-9}$ Pa. The high-resolution XPS spectra were recorded using 100 meV per step.

### 2.6 Optical characterization

UV-Vis spectra were collected using a JASCO V-630 spectrophotometer between 200 and 1100 nm (scanning at 400 nm min^−1^) using diluted particle dispersions in toluene. Photoluminescence (PL) and photoluminescence excitation (PLE) were acquired using a Shimadzu spectrofluorophotometer model RF-6000. The CdSe photoluminescence quantum yields ($\Phi_{\text{QY}}$) were calculated by comparing with Rhodamine 6G emission ($\Phi_{\text{6G}}$ = 95%, ethanol solution) keeping the excitation wavelength lower than 0.05. The integrated PL spectra of CdSe samples ($I_{em,QD}$) and Rhodamine 6G ($I_{em,6G}$) are used to calculate the $\Phi_{\text{QY}}$:

(1) $$\Phi_{\text{QY}}=\frac{1-10^{Abs_{6G}}}{1-10^{Abs_{CdSe}}}\frac{I_{em,QD}}{I_{em,6G}}\frac{n^{2}}{n_{6G}^{2}}\Phi_{\text{6G}}$$

where *Abs* is the optical density in the absorption spectra at the excitation point, and *n* is the refractive indices of solvents.

Jasieniak and coworkers proposed methodology was used to calculate the size and size distribution of CdSe QDs from UV-Vis data [36].

(2) $$\bar{D}(E_{1S})=59.60816-0.54736\lambda_{1}+1.8873\times 10^{-3}\lambda_{1}^{2}\;\;-2.85743\times \times 10^{-6}\lambda_{1}^{3}+1.62974\times 10^{-9}\lambda_{1}^{4}$$

(3) $$\bar{\sigma }=\frac{D(E_{1S}+\Delta E_{1S})-D(E_{1S}-\Delta E_{1S})}{\bar{D}(E_{1S})}$$

where $\bar{D}(E_{1S})$ is the average diameter calculated from the position of the first excitonic absorption band ($1S_{e}-1S_{h}$, $E_{1S}$), $\lambda_{1}$ is the wavelength value (nm) of $E_{1S}$ maximum absorption, $\Delta E_{1S}$ is half-width at half-maximum (FWHM) of the low-energy tail of the first absorption band, and $\bar{\sigma }$ is a percent estimation of the particle size distribution by the width from $1S_{e}-1S_{h}$ band. The time-dependent growth kinetics study was realized after the R–O–SeOOH injection (0 s), quickly collecting 100 μL of the reaction media and injecting it in 2 mL of cold toluene (ice bath) to quench QDs growth. Other eight aliquots were withdrawn after 15, 30, 60, 90, 120, 180, 300, and 420 s.

Luminescence decays of the CdSe QDs in toluene solution were measured by time-correlated single-photon counting technique (TCSPC) using a Peltier-cooled PMT-MCP (Hamamatsu R3809U-50) as a photon detector. Light pulses at 400 nm were provided by frequency doubling the 150 fs laser pulse of a Ti-Sapphire Mira 900 laser pumped by Verdi 5 W (Coherent). The emission decays at 520 nm were collected in magic-angle mode (54.7°) using Glan-Laser polarizers (Newport), with $4\times 10^{3}$ peak counts, time increment of 100-200 ps per channel recorded with the TC900 counting board from Edinburgh Instruments.

## 3 Results and discussion

**Figure 1:**
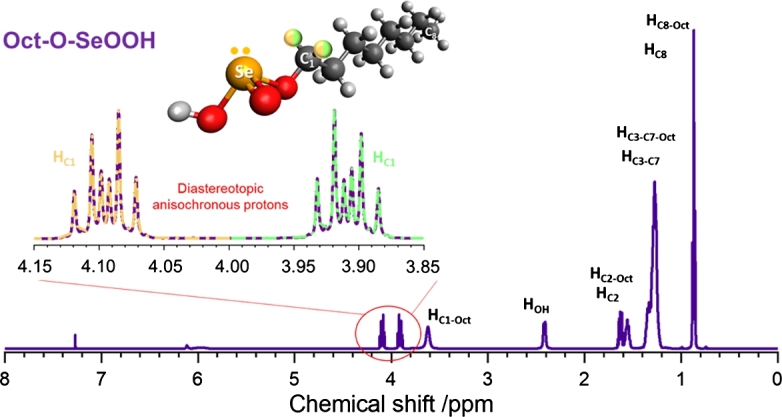
^1^H NMR spectrum of the reaction product from  with Oct–OH. The inset shows the signal from the diastereotopic protons (hydrogen colored orange/green on ), showing the anisochronous chemical shift (splitting of peaks onto two regions around 3.9 and 4.1 ppm). Based on the Se chiral environment, these signals infer the formation of alkyl acid selenites (Oct–O–SeOOH). The structure of the Oct–O–SeOOH molecule is also shown, highlighting the diastereotopic protons. Concerning the stoichiometric ratio :R–OH, some residual signals from pure (excess) alcohol can be observed in the spectrum, identified with a subscript of the corresponding carbon chain (*e.g*., H${}_{C8-Oct}$).

 reacts with alcohol molecules (R–OH) at ordinary temperatures leading to a colorless solution [33], [34], [35]. However, this reaction has never been explored to obtain selenium precursors for QDs syntheses. Here, the solubility of  in several R–OH was evaluated, leading to a wide range of Se precursors for CdSe QDs synthesis, see Fig. S1 in the Supporting Information (SI). We choose three samples to discuss the effectiveness of these precursors in QDs synthesis: an alcohol with the smallest C-chain (methanol, Met–OH), an intermediate size alkyl chain (1-octanol, Oct–OH), and a long-chain fatty alcohol (oleyl alcohol, Oleyl–OH). All  solutions were kept at 2.0 mol L^−1^. The chemical structure of the reaction products between  and R–OH was investigated by ^1^H NMR. Fig. 1 shows a representative spectrum for the reaction product of  with Oct–OH. Figs. S2-6 in the SI show other ^1^H NMR results, including the spectra of pure R–OH molecules.

**Scheme 1:**
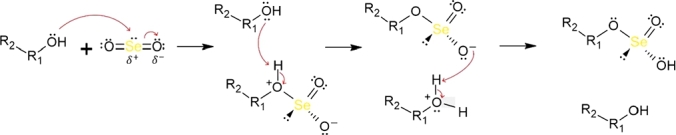
Chemical reaction of an alcohol molecule (R–OH) with selenium dioxide leading to alkyl acid selenites (R–O–SeOOH) and their resonance contributors.

All ^1^H NMR spectra show the reference (TMS) and  peaks with chemical shifts *δ* = 0 ppm and *δ* = 7.26 ppm, respectively. As excess of R–OH was used to prepare the precursor, stoichiometry :R–OH was not 1:1, and the signals of pure alcohol are present in the ^1^H NMR spectra (see Fig. S2 for pure Oct–OH). The remaining peaks were assigned for the reaction product, which showed a slightly deshielded chemical shift than the pure alcohol spectrum, *i.e.*, higher *δ* values were observed. The deshielded signal indicates the proximity with the Se atom, which is more electronegative than the oxygen of alcohol's hydroxyl group. Although these slight variations in the *δ* values can be identified, no significant change in multiplicity was observed, indicating that the hydrocarbon chain remains unchanged. In the analysis at the region between 3.85 ppm and 4.15 ppm, where the H${}_{C1}$ signal is expected, the ^1^H NMR signal of the anisochronic diastereotopic protons with complex multiplicity was observed. The presence of the two double triplets signals, one between 3.85 and 3.95 ppm (green double triplets) and another from 4.0 to 4.15 ppm (orange double triplets) proves the existence of a chiral environment around the Se atom, Fig. 1. These complex signals occur due to the different magnetic environments around the H${}_{C1}$ protons induced by the Se chiral center (C_1_–SeOOH). The multiplicity (triplet) occurs from the coupling of the two protons of the neighbor methylene group (), and the split of this triplet in the two double triplets appears due to the coupling with the other H${}_{C1}$. Although these H${}_{C1}$ protons have different chemical environments, it is practically impossible to differentiate them; thus, the H${}_{C1}$ protons were represented as half orange and half green. Selenium chirality has already been reported in the literature [37]. The tetrahedron Se structure with four different ligands, including the lone pairs, generates a chiral environment. This chiral environment explains the diastereotopic anisochronous protons signal, inferring that  is closest to Selenium. ^1^H NMR results suggest that  is bound to oxygen from the hydroxyl group of the R–OH molecules, *i.e.*, R–O–SeOOH. A molecular representation of the compound is displayed in Fig. 1 [33].  solubility occurs via a reaction with R–OH molecules to form R–O–SeOOH according to the proposed mechanism in Scheme 1 [33], [34], [35]. The oxygen from the R–OH molecule is a Lewis base that acts as a nucleophile attacking the Se atom, which has a partial positive charge, thus is formed a zwitterionic species that, after a process of proton transfer, results in the formation of acid selenite.

The reaction mechanism proposed for R–O–SeOOH formation agrees with the ^1^H NMR spectra of precursors synthesized using other alcohols, Figs. S2-6. For Met–OH, as only one methyl (–) is present, ^1^H NMR spectra show just the presence of an extra peak compared to pure alcohol due to the slightly deshielded – group in the presence of Se, Fig. S3. In the case of isopropanol, the  has just one proton, and no diastereotopic behavior was observed as expected. Instead, a septet occurred due to the coupling with protons of the methyl groups of  and  (Fig. S6).

**Figure 2:**
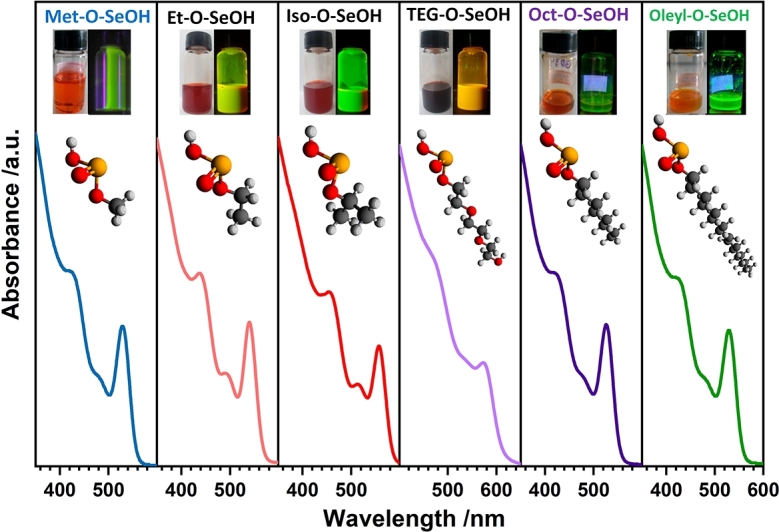
UV-Vis spectra of CdSe QDs synthesized using (from left to right): methanol (Met—OH), ethanol (Et—OH), isopropanol (Iso—OH), triethylene glycol (TEG—OH), 1-octanol (Oct—OH), and oleyl alcohol (Oleyl—OH).

For all other alcohols, the ^1^H NMR spectra show diastereotopic protons signals, Figs. S4-5, confirming the formation of the acid selenite structures. Therefore, ^1^H NMR results prove that R—O—SeOOH compounds can be prepared with a wide range of R–chains, leading to several new Se precursors for CdSe QDs synthesis. The samples of colloidal CdSe Qds obtained from these new Se precursors are displayed in Fig. 2, showing the new precursor's efficiency and versatility. Table S1 summarize the UV-Vis features for all samples including their luminescence quantum yields ($\Phi_{\text{QY}}$).

**Figure 3:**
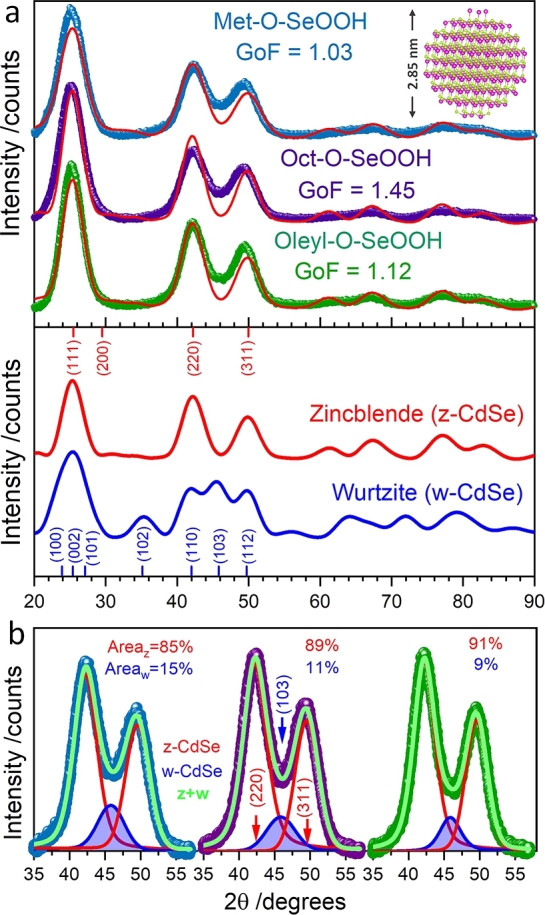
(a) XRD patterns of CdSe samples synthesized using Met–O–SeOOH, Oct–O–SeOOH, Oleyl–O–SeOOH selenium precursors compared to simulated X-ray scattering pattern for the cubic (zinc blende, $F\bar{4}$3m) and hexagonal (wurtzite, *P*6_3_*mc*) phases. (b) Deconvolution of the XRD peaks between 35° and 55°, showing an additional reflection (blue area) corresponding to the (103)_*w*_ diffraction from the w-CdSe structure.

XRD patterns for CdSe QDs obtained using the Met–O–SeOOH, Oct–O–SeOOH, and Oleyl–O–SeOOH molecules are displayed in Fig. 3. Simulated X-ray scattering patterns for zinc blende (z-CdSe, $F\bar{4}3m$, ICSD 41528, red curve) and wurtzite (w-CdSe, $P6_{3}mc$, ICSD 41491, blue curve) structures are also shown, highlighting their main peaks positions and Miller indexes. All three samples showed similar XRD results expected for small nanocrystal sizes, which lead to elevated peak broadening, *i.e.*, the absence of defined Bragg peaks and the presence of diffuse scattering. Only short- (SRO) and medium-range order (MRO) are present in QDs, making the structure analysis hard. Besides, nanoparticles also have size broadening from size effects like structure relaxation, surface atomic arrangement, and stacking faults defects [38]. Debye equation is an alternative method to calculate the XRD patterns of QDs and fit experimental data to accomplish the structure determination according to Equation (4).

(4) $$I(Q)=\sum_{i}\sum_{j}f_{i}f_{j}\frac{\sin(Qr_{ij})}{Qr_{ij}}$$

where $f_{i}$ and $f_{j}$ are the atomic scattering factors for all possible atomic pairs within the structure, $r_{ij}$ is the distance between atoms *i* and *j*, *Q* is the magnitude of the reciprocal space wave vector (Q=4πsin⁡(θ)/λ), 2*θ* is the angle between the incident and scattered X-rays, and *λ* is the X-rays wavelength.

Experimental CdSe QDs patterns were indexed to the z-CdSe phase when compared to simulated XRD patterns. Using the software Debussy 2.0 [39], the patterns could be fitted using a size distribution XRD model, which considers the XRD signal contribution for previous simulated nanocrystals structure ranging from 1 to 5 nm, see Fig. S7. The first three main peaks at 25.1°, 42.2°, and 49.5°in 2*θ* were indexed to (111)_*c*_, (220)_*c*_, and (311)_*c*_ diffraction planes. The average size and standard deviation values obtained by the XRD fitting (${\bar{D}}_{XRD}$) for samples Met–O–SeOOH, Oct–O–SeOOH, and Oleyl–O–SeOOH were 2.7±0.35 nm, 2.8±0.30 nm, and 3.0±0.50 nm, respectively. These results corroborate the calculated crystallite size by the Scherrer equation using the (111)_*c*_ peak of *ca.* 3 nm for all samples. The strain and thermal vibrations (Debye-Waller factor) were accounted for using Debussy software, but nanocrystals also present other sources of size broadening previously mentioned that were not considered here [38]. Fig. 3 inset shows the structure of a 2.8 nm spherical z-CdSe particle with nine stacking sequences along the [111]_*c*_ direction, demonstrating the mean structure model obtained by the fitting analysis. However, pure z-CdSe does not fit perfectly the XRD results. The deviations between the simulated and the experimental data indicate the presence of defects in the CdSe structure. The region around the (220)_*c*_ and (311)_*c*_ reflections presents discrepancies that match the position of the (103)_*w*_ from the w-CdSe structure in Fig. 3b. The deconvolution of the XRD peaks between 35°to 55°reveals the presence of a third reflection (blue area) indexed to the (103)_*w*_ diffraction peak from w-CdSe. The peak area for the w-CdSe phase decreases with the increase of R–O–SeOOH alkyl length, which corresponds to 15% (Met–O–SeOOH), 11% (Oct–O–SeOOH), and 9% (Oleyl–O–SeOOH) of the total area. This result indicates that the alkyl length in the acid selenite precursor can control the number of defects in CdSe nanoparticles.

CdSe QDs structures (z-CdSe and w-CdSe) are polymorphs, where the cubic phase (z-CdSe) is thermodynamically favorable at low temperatures with phase transition around 100 °C for bulk CdSe [40]. The energy difference between both structures is around 1.4 meV/CdSe unit [40]. This relatively low energy allows the formation of different stacking sequences during the synthesis leading to both crystallographic profiles within the nanocrystal, a crystallographic defect known as stacking faults [41]. The z-CdSe has a stacking sequence ABCABC in the [111]_*c*_ direction, while the w-CdSe has an ABABAB stacking sequence along the [001]_*w*_ direction [40]. Although z-CdSe corresponds to the majority structure observed in XRD results, hexagonal stacking sequences are also observed (ABA, w-CdSe). The presence of hexagonal stacking sequences contributes to the reflection (103)_*w*_ appearance at 45.8°, which increases the signal between the (220)_*c*_ and (311)_*c*_ peaks. At high-temperature synthesis (> 200 °C) the formation of the w-CdSe would be expected. However, materials at the nanoscale can display metastable phases, especially in colloidal synthesis, where several molecules interact with the surface of nanocrystals (solvent, ligands, precursors, and others). Gao and colleagues have shown that the cadmium-ligand coordination nature is a crucial factor in the final nanocrystal structure, where cadmium-phosphonate ligands lead to w-CdSe and cadmium–carboxylate ligands to z-CdSe [40]. Even though the literature shows that z-CdSe or w-CdSe occurrence depends on the ligands [42], [43], the selenium precursor effect is not well known. Here, the z-CdSe with stacking fault was observed, and using a longer alkyl chain leads to a lower concentration of defects. To better understand the structure of the samples, HRTEM images will be discussed later.

**Figure 4:**
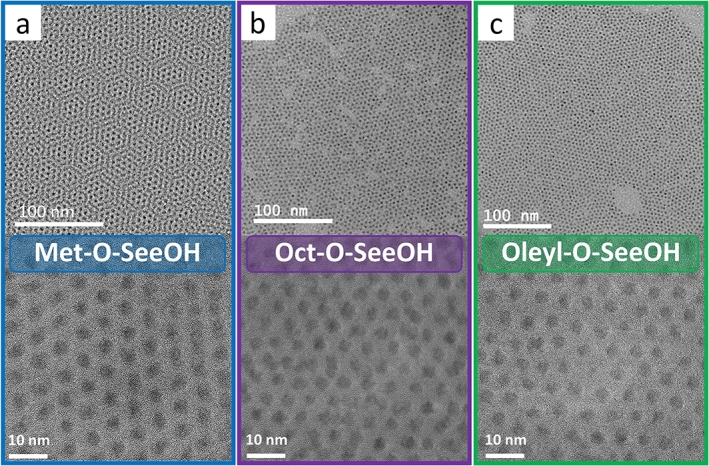
TEM images of CdSe QDs samples synthesized using (a) Met–O–SeOOH, (b) Oct–O–SeOOH, and (c) Oleyl–O–SeOOH precursors.

Monodisperse spherical CdSe QDs are obtained using Met–O–SeOOH, Oct–O–SeOOH, and Oleyl–O–SeOOH precursors, Fig. 4. Here, no size-selective precipitation was used, *i.e.*, the obtained product is the as-synthesized nanomaterial. Two different magnifications are presented in Fig. 4 to show the morphology control obtained using acid selenite precursors. The nanoparticles produced using Met–O–SeOOH precursor showed smaller sizes (${\bar{D}}_{TEM}=2.7$ nm) and the narrowest size distribution (σ=0.3 nm), Fig. 4a. Oct–O–SeOOH (Fig. 4b) and Oleyl–O–SeOOH (Fig. 4c) samples showed ${\bar{D}}_{TEM}$ equal 3.2±0.41 nm and 3.7±0.44 nm, respectively. The histograms for the three samples are displayed in the supporting information, Fig. S8. Oleic acid-capped CdSe QDs showed self-assembly behavior in the TEM grid, reflecting the isotropic van der Waals interaction between particles (monodisperse sample), as shown in the hexagonal superlattice pattern observed in Fig. 4a.

Based on the classical nucleation and growth theory, to prepare monodisperse nanocrystals, the precursor must be highly reactive to control the separation of these two processes [25]. It was observed that acid selenites are highly reactive, leading to the initial burst nucleation necessary to synthesize monodisperse nanoparticles. Although alcohol molecules can also act as ligands, the small-chain molecules have lower boiling points and evaporate from the reaction medium promptly after injection. The long-chain alkyl ligands as Oleyl–O–SeOOH remain in the reaction medium, but no significant differences in the final nanoparticle size were noted. Also, oleic acid was used to control nanoparticle size as it binds more strongly to the CdSe surface than alcohol. The soft ligand oleylamine was added to assist the selenium reduction along with 1,2-hexadecanediol. All the acid selenite used here showed significant size control for CdSe synthesis, Fig. 2. Besides the CdSe synthesized in the quantum confinement regime (< 5.6 nm) [44], TEG–O–SeOOH (the higher viscosity solution) leads to slow solubilization making the formation of smaller nanocrystals with green emission difficult.

**Figure 5:**
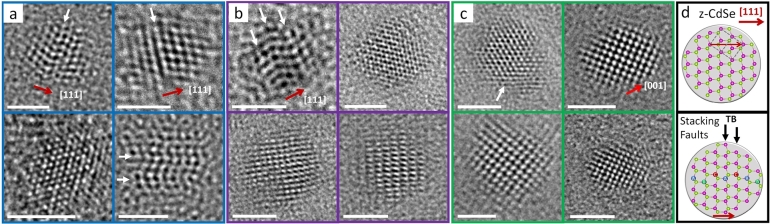
HRTEM images of individual CdSe QD for samples (a) Met–O–SeOOH, (b) Oct–O–SeOOH, and (c) Oleyl–O–SeOOH precursors. The [111]_*c*_ direction (red arrows) is indicated along with the presence of twin boundary defects (TB, white arrows) originated by stacking faults. The scheme in (d) shows a 2.8 nm CdSe QD displaying the atom position, the cubic unit cell, and the [111]_*c*_ direction. The scheme in (c) shows a stacking fault defect leading to both z-CdSe (ABC) and w-CdSe (ACA) stacking sequences indexed along with the TB position.

HRTEM images of Met–O–SeOOH, Oct–O–SeOOH, and Oleyl–O–SeOOH samples are displayed in Fig. 5a-c. The inset in Fig. 5d (above) shows a 2.8 nm CdSe nanocrystal model in the $\left[1\bar{1}0\right]$ zone axis displaying a cubic unit cell with the [111]_*c*_ direction indicated by the red arrow, assisting the HRTEM images discussion. Likewise, the second nanocrystal model presented in Fig. 5 (below) shows a stacking fault defect and the formation of a twin boundary-type (TB) defect. The disruption of the SRO from the pristine z-CdSe phase (ABCABC) to a TB zone can be visualized using HRTEM in a specific zone axis, which appears as a zigzag shape in the [111]_*c*_ direction. HRTEM images of the pure w-CdSe phase were not found. For all three samples, crystalline spherical nanoparticles can be seen. It should be noted that the HRTEM analysis is a highly local characterization technique and cannot be used to infer statistical conclusions about the defect population in this case. However, the individual QDs set analyzed and represented by the images in Fig. 5 agree with the XRD data, considering the deconvolution of peaks (220)_*c*_ and (311)_*c*_ with the (103)_*w*_ from w-CdSe phase. Our results showed that an increase in the alkyl chain from the different acid selenite precursors leads to decreased structural defects in QDs. Thus, the number of defects follows Met–O–SeOOH > Oct–O–SeOOH > Oleyl–O–SeOOH, suggesting that this behavior can be extrapolated to the other precursors.

**Figure 6:**
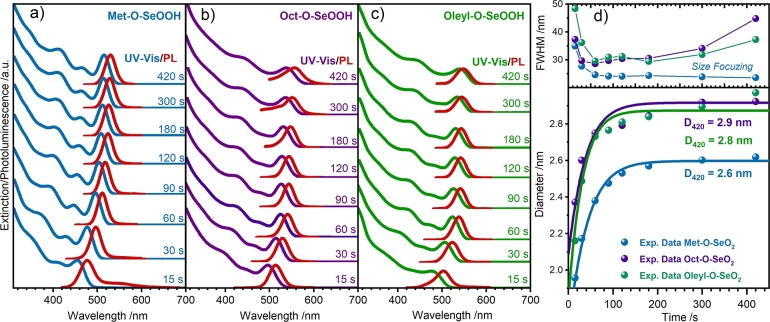
Normalized absorbance and photoluminescence (PL) spectra for CdSe samples (a) Met–O–SeOOH, (b) Oct–O–SeOOH, and (c) Oleyl–O–SeOOH showing the growth time evolution based on the position of the first exciton (1*S*_*h*_ − 1*S*_*e*_) band *λ*_1*st*_. (d) The time evolution kinetics is summarized in the time versus size graphic and the full-width half maximum (FWHM) time evolution of the first excitonic peak. An exponential curve line is shown as a guide to the eye.

Time evolution UV-Vis spectra of CdSe samples are shown in Fig. 6, along with their respective photoluminescence (PL) spectra. The excitonic bands correspond to $1S_{h}-1S_{e}$ (first exciton band $\lambda_{1st}$), $1P_{h}-1P_{e}$, $1D_{h}-1D_{e}$, and $2S_{h}-2S_{e}$ transition can be observed for Met–O–SeOOH sample, indicating size-controlled nanocrystals obtained here. At least three excitonic bands can be seen for the other samples. After R–O–SeOOH injection (0 s), the aliquots were taken from 15 s to 420 s growth. The average QDs size obtained by the position of $\lambda_{1st}$ bands ($\bar{D}(E_{1S})$) was calculated using Jasieniak and coworkers method (see Equation (2)) [36]. The $\lambda_{1st}$ band and, consequently $\bar{D}(E_{1S})$, changes with the R–O–SeOOH chain size.

The UV-Vis allows us to evaluate the size distribution concerning the FWHM of the $\lambda_{1st}$ bands, Fig. 6d. The size distribution observed by UV-Vis analysis for samples Oct–O–SeOOH and Oleyl–O–SeOOH increased after 200 s of growth step. Only the sample Met–O–SeOOH presented a size-focusing regime. Small alkyl chains () have less stability at high temperatures than  or , leading to faster selenium availability after precursor injection. As observed for Met–O–SeOOH precursor, other small carbon chains alcohol molecules like ethanol and isopropyl alcohol generate better selenium precursors to synthesize small-sized CdSe QDs (data not shown). Most of the precursor molecules are consumed during nucleation, so the reaction reaches a fast equilibrium and the final size distribution is narrowed. Compared to the classical TOP-Se precursors, R–O–SeOOH precursors are similar to TOP-Se reactivity as the growth kinetics was observed in the same time range (0-300 s) as shown by Bullen and Mulvaney [26].

The average diameters from TEM ${\bar{D}}_{TEM}$ were compared with the average crystallite size from XRD (${\bar{D}}_{XRD}$) and with the estimated diameter from UV-Vis ($\bar{D}(E_{1S})$), Fig. S9. It is possible to establish that all results lead to a good correlation for controlled-shaped spherical CdSe QDs. The values were evaluated considering an error of three times the standard deviations (99.73% of the population) from ${\bar{D}}_{TEM}$. The Oleyl–O–SeOOH sample showed a higher variance than the Met–O–SeOOH ones. This comparison allows inferring that the estimated sizes (${\bar{D}}_{XRD}$ and $\bar{D}(E_{1S})$) are consistent with the morphological diameter by TEM (${\bar{D}}_{TEM}$) and, despite the stacking faults, the CdSe QDs possess a small number of crystallographic defects. Comparing the CdSe QDs synthesized using Met–O–SeOOH with the standard TOP-Se methods [7], it is possible to conclude that the size distribution of QDs (optical emission purity) is comparable. As reported by Wu and colleagues, the distance between the two first excitonic absorption bands $1S_{e}-1S_{h}$ and $1S_{e}-2S_{h}$ is higher for z-CdSe [45], confirming the cubic structure of the CdSe QDs reported here (Fig. S10).

**Figure 7:**
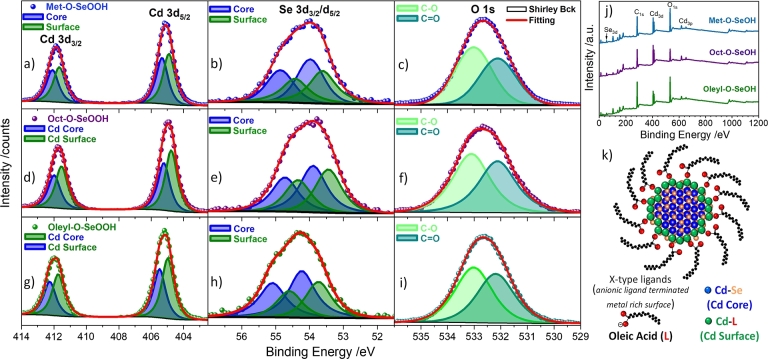
High-resolution XPS spectra of Cd 3*d* region for (a) Met–O–SeOOH, (b) Oct–O–SeOOH, and (c) Oleyl–O–SeOOH samples showing the deconvolution of 3*d*_5/2_ and 3*d*_3/2_ attributed to cadmium bound to selenium at the bulk (Cd–Se, blue) and to oxygen at the surface (Cd–O, green). (d) A schematic representation of a CdSe QD with oleic acid at the surface shows the Cd–Se (blue atoms) within the bulk and the Cd–O (green atoms) at the surface, matching the XPS Cd 3*d* bands deconvolution indexation.

XPS survey and high-resolution analysis for Cd 3*d*, Se 3*d*, and O 1*s* of the three samples are shown in Fig. 7, Table S2 presents the result parameters. The samples were freshly synthesized CdSe QDs with minimal exposure to air as synthesis and washing procedures were performed in  and moisture-controlled environments. The Cd 3*d* signal is composed of two sets of split bands assigned to: Cd–Se bound (core, blue) located at 405.34 eV ($d_{5/2}$) and 412.11 ($d_{3/2}$), and a second split band's contribution at 404.88 eV ($d_{5/2}$) and 411.65 ($d_{3/2}$), which was attributed to Cd chemical environment at the QDs surface (green). The indication of core level for CdSe QDs was done using the position of Cd $3d_{5/2}$ band position range (405.1 eV to 406 eV) [46]. No Cd–O oxygen band was observed for the samples in the expected band position region *ca*. 404.2 eV. In addition, the O 1*s* spectra do not show any structural metal oxide signals expected in the 529 to 530 eV range. The only deconvolutions observed in the O 1*s* spectra were attributed to molecular C–O and C=O from oleic acid. The XPS spectra of the Se 3*d* for all samples are similar, indicating one or two species that are difficult to distinguish due to the small split from spin-orbit components. Here two species were used to support the contribution of both structure Cd–Se from the core and the disordered chemical environment at the surface. The survey shows a high content of carbon (C 1*s*) and oxygen (O 1*s*) from the ligands onto the surface of nanoparticles, as expected for small quantum dots. The relative proportions of Cd:Se were similar for all samples, 60:40 (Met–O–SeOOH), 59:41 (Oct–O–SeOOH), and 64:36 (Met–O–SeOOH), indicating a Cd-rich surface, see Table S2. This is expected since an anionic X-type ligand (oleate ions from oleic acid molecules) coordinates with the metal atom (Lewis acid) at the surface of nanoparticles, Fig. 7k. Also, no N 1*s* bands were observed around 400 eV related to amines (R–NH_2_), showing that oleylamine molecules mainly were washed during the nanoparticle's purification process.

**Figure 8:**
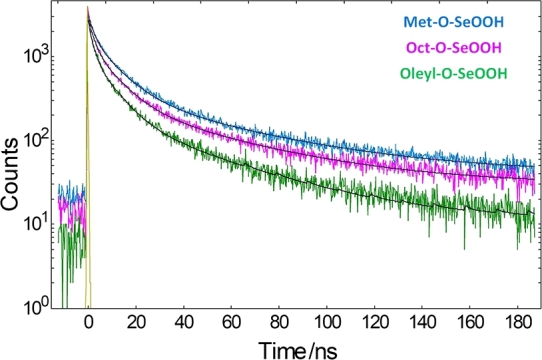
PL decays for Met–O–SeOOH (blue curve), Oct–O–SeOOH (red), and Oleyl–O–SeOOH (green) samples.

The PL lifetimes for CdSe QDs samples are shown in Fig. 8. Decay components were evaluated by deconvolution procedure with multiexponential decay models (Equation (5)), where triexponential fitting parameters were used to express the PL decay.

(5) $$I(t)=\sum_{i=1}A_{i}\exp(-t/\tau_{i})$$

where $\tau_{i}$ and $A_{i}$ are the decay time and pre-exponential factor of the $i^{th}$ component, respectively.

All measured luminescence decays were well described with three decay components, as reported in Table 1. Recorded luminescence decays and fitting results show a multiexponential relaxation of the electronically excited state CdSe QDs (ensemble sample) in toluene suspension, as illustrated in Fig. 8. The major luminescence decay component is the fast one with a value of the order of 2 ns. However, the weighted average lifetime <*τ*> is controlled by the long decay contributions correlated to nonradiative rate transitions, leading to lower emission efficiency [47]. Then, <*τ*> values can correlate with the sample's luminescence quantum yields ($\Phi_{\text{QY}}$). We have observed that the $\Phi_{\text{QY}}$ for the R–O–SeOOH samples increase with the R-chain length. $\Phi_{\text{QY}}$ was lower for sample Met–O–SeOOH (2%), increasing for samples Oct–O–SeOOH (5%) and Oleyl–O–SeOOH (15%). The opposite occurred for the <*τ*> values showing 25.2 ns for Met–O–SeOOH, 22.2 ns for Oct–O–SeOOH, and faster decay of 18.5 ns for Oleyl–O–SeOOH, *i.e.*, Oct–O–SeOOH and Oleyl–O–SeOOH samples have higher $\Phi_{PL}$ while sample Met–O–SeOOH is less emissive due to higher contribution of nonradiative rate transitions. The quantum yield values reported here agree with those reported for bare  QDs, *i.e.*, without surface coating layers. In these cases, trap states located in the surface of CdSe due to the dangling bonds lead to longer PL lifetimes and low QY values [48]. As all CdSe handling was the same and special care was taken to keep the same washing procedure, the $\Phi_{\text{QY}}$ results indicate that the R–O–SeOOH precursor also influences the CdSe QDs optical properties. As Oleyl–OH has been used as a surfactant for some nanoparticles synthesis, it is possible that its acid selenite compound and the excess of Oleyl–OH in the injection solution could also act as co-surfactant contributing to controlling the CdSe surface and lowering the number of trap states. Consequently, Oleyl–O–SeOOH present higher PL QY compared to small chain R–O–SeOOH precursors that are lost at higher temperature synthesis due to evaporation.

**Table 1:** Luminescence decay time (*τ*), normalized amplitude (*A*), and average lifetime (<*τ*>) for CdSe QDs samples Met–O–SeOOH, Oct–O–SeOOH, and Oleyl–O–SeOOH.

Sample	*τ*_1_/ns (*A*_1_)	*τ*_2_/ns (*A*_2_)	*τ*_3_/ns (*A*_3_)	<*τ*>/ns
Met–O–SeOOH	2.0 (0.66)	10.0 (0.28)	49.6 (0.06)	25.2
Oct–O–SeOOH	2.0 (0.62)	9.4 (0.32)	45.5 (0.06)	22.2
Oleyl–O–SeOOH	1.7 (0.68)	8.4 (0.27)	39.7 (0.05)	18.5

## 4 Conclusions

Alkyl acid selenites (R–O–SeOOH) were readily and efficiently synthesized from the reaction of  with alcohol molecules (R–OH) and used as selenium precursors for CdSe QDs synthesis. NMR results indicate that the R–O–SeOOH molecules are successfully obtained, resulting in the Se source during QDs synthesis. This new Se precursor can generate high-quality and monodisperse CdSe nanocrystals, demonstrating that R–O–SeOOH molecules are highly reactive at high temperatures. Our initial study of the CdSe growth indicates that burst nucleation occurred shortly after precursor injection. Thus, the classical separation of nucleation and growth regimes to produce homogeneous nanocrystals was achieved using the proposed synthetic route (hot injection of R–O–SeOOH molecules), successfully resulting in a narrow nanoparticle size distribution. Six different alcohols were used to study the CdSe QDs morphology, structure, and properties, as shown for the methanol (R = ), 1-octanol (R = ), and oleyl alcohol (R = ) samples. The results prove that R–O–SeOOH molecules lead to high-quality CdSe QDs comparable to the standard phosphine TOP-Se precursor protocol. Our findings also allow exploring other synthetic systems (Cd precursor, solvents, ligands, among others) together with the R–O–SeOOH precursors. The  has high chemical stability, and alcohol compounds are cheap and less toxic than standard reactants used in CdSe QDs synthesis. Thus, the R–O–SeOOH compounds have the potential to be a cost-competitive selenium source for synthesizing CdSe and other metal chalcogenides nanomaterials. Therefore, the alkyl selenite molecule discussed here is an efficient and easy Se precursor for monodisperse CdSe QDs synthesis.

## CRediT authorship contribution statement

**João B. Souza Junior:** Conceptualization, Data curation, Formal analysis, Funding acquisition, Investigation, Methodology, Writing – original draft, Writing – review & editing. **Beatriz Mouriño:** Formal analysis, Investigation, Methodology, Writing – original draft, Writing – review & editing. **Marcelo H. Gehlen:** Data curation, Formal analysis, Investigation, Writing – original draft, Writing – review & editing. **Daniel A. Moraes:** Data curation, Formal analysis, Investigation, Methodology, Writing – original draft, Writing – review & editing. **Jefferson Bettini:** Data curation, Formal analysis, Investigation, Writing – original draft, Writing – review & editing. **Laudemir C. Varanda:** Conceptualization, Data curation, Formal analysis, Funding acquisition, Investigation, Methodology, Project administration, Supervision, Writing – original draft, Writing – review & editing.

## Declaration of Competing Interest

The authors declare that they have no known competing financial interests or personal relationships that could have appeared to influence the work reported in this paper.

## Data Availability

The original data have been uploaded to the Zenodo platform (https://doi.org/10.5281/zenodo.10229635), and more detailed data content can be requested from the corresponding authors.

## Appendix A Supplementary material

MMC Supplementary material includes additional acid selenites ^1^H NMR characterization, CdSe QDs histogram of counting, and additional optical characterization. MMC
