# Supplementary material for: Acid selenites as new selenium precursor for CdSe quantum dot synthesis
Source: Heliyon. 2023 Dec 15;10(1):e23837. doi: 10.1016/j.heliyon.2023.e23837 (PMC10777003; doi:10.1016/j.heliyon.2023.e23837)
Supplement: MMC — Supplementary material includes additional acid selenites 1H NMR characterization, CdSe QDs histogram of counting, and additional optical characterization. [file mmc1.pdf]

Acid Selenites as New Selenium Precursor for CdSe Quantum Dot Synthesis

João Batista Souza Junior,[1,2] Beatriz Mouriño,[1] Marcelo Henrique Gehlen,[1] Daniel Angeli de Moraes,[1,2] Jefferson Bettini,[2] and Laudemir Carlos Varanda[1]

[1]*Instituto de Química de São Carlos, Universidade de São Paulo – USP, Colloidal Materials Group, CP 780, 13566-590, São Carlos – SP, Brazil*

joao.junior@lnnano.cnpem.br; lvaranda@iqsc.usp.br

[2]*Brazilian Nanotechnology National Laboratory (LNNano), Brazilian Center for Research in Energy and Materials (CNPEM), 13083-970, Campinas, Brazil*

Supporting Information

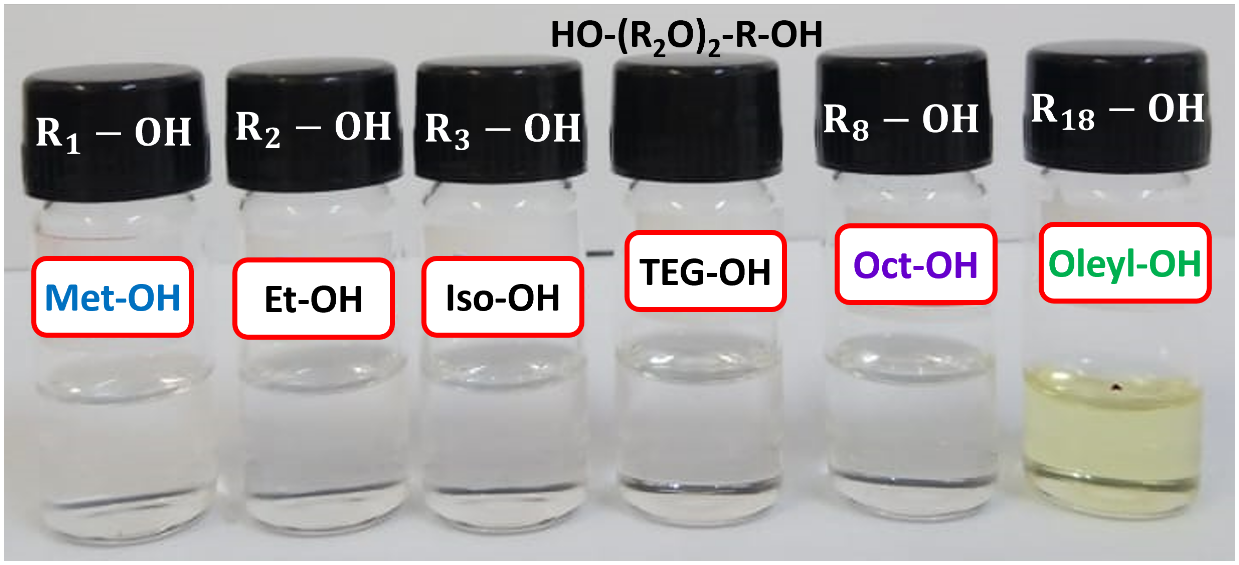

FIG. S1. Photograph of colorless reaction product of SeO_2 with several alcohols (R–OH), from left to right, methanol (Met–OH), ethanol (Et–OH), isopropanol (Iso–OH), triethylene glycol (TEG–OH), octanol (Oct–OH), and oleyl alcohol (Oleyl–OH).

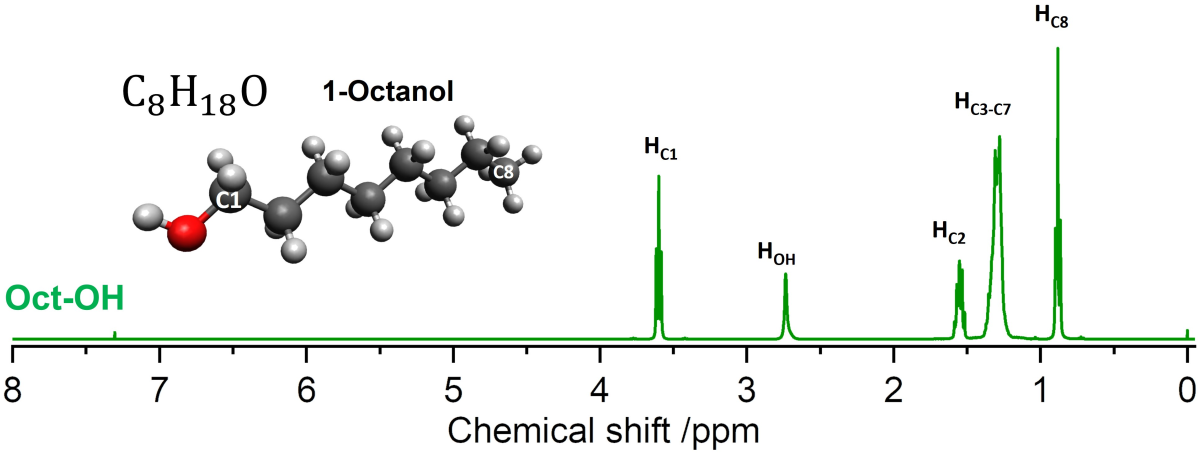

FIG. S2. ^{1}H NMR spectrum of 1-octanol (Oct–OH) alcohol.

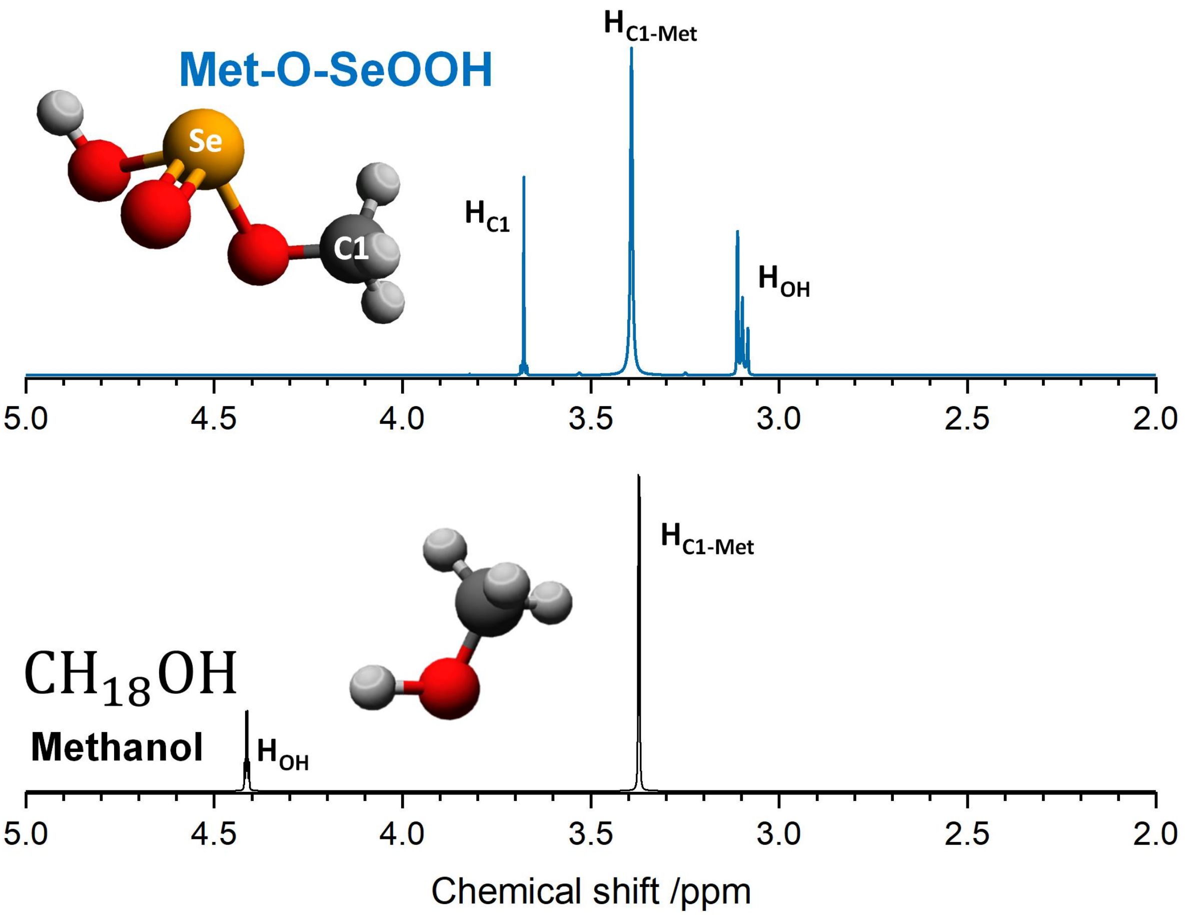

FIG. S3. ^{1}H NMR spectrum for the reaction product of SeO_2 with methanol (Met–OH) leading to Met–O–SeOOH along with the spectrum of pure methanol.

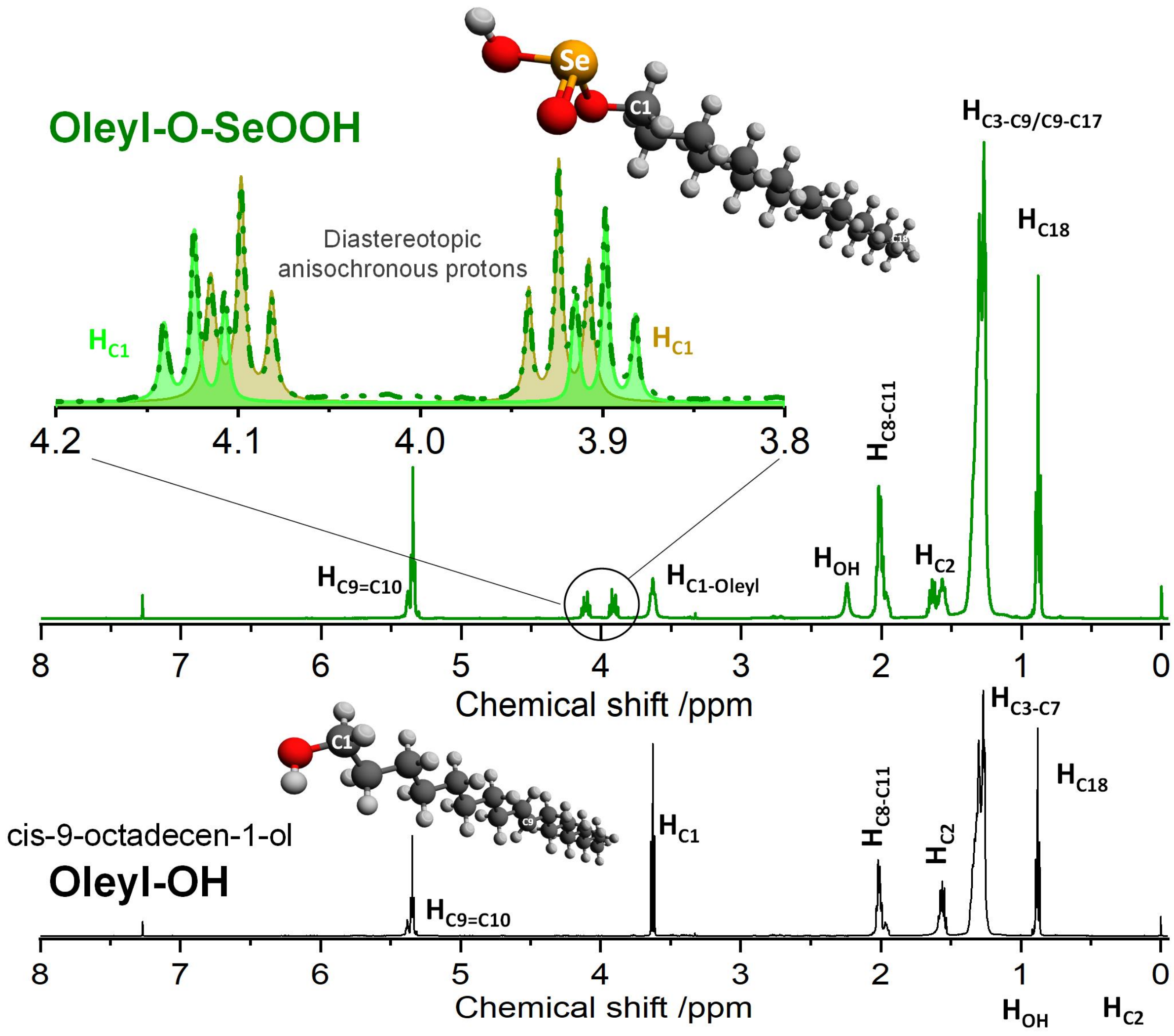

FIG. S4. ^{1}H NMR spectrum for the reaction product of SeO_2 with oleyl alcohol (Oleyl–OH) leading to Oleyl–O–SeOOH along with the spectrum of pure Oleyl–OH.

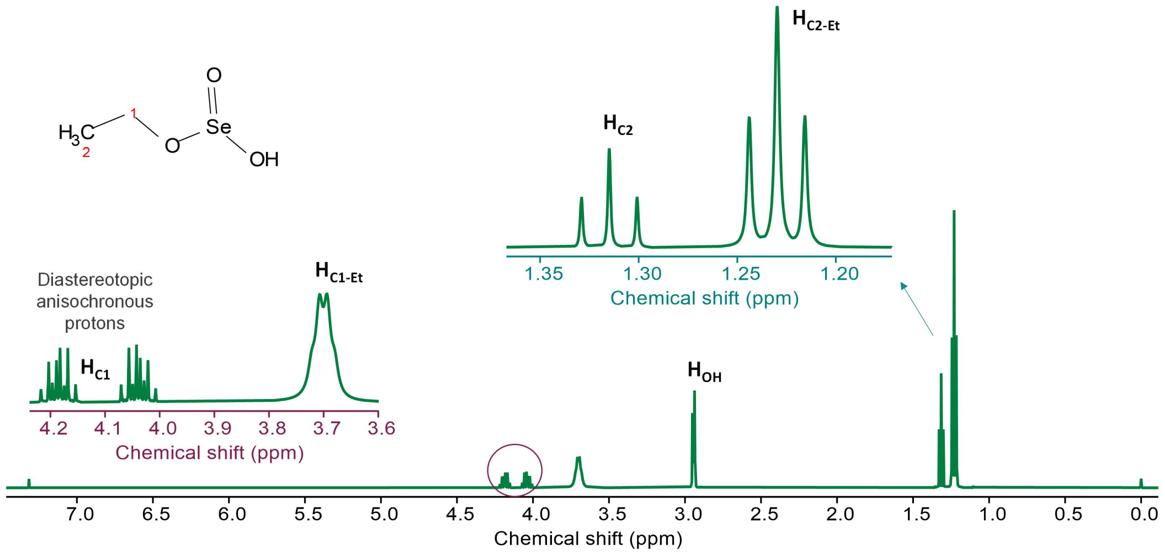

FIG. S5. ^{1}H NMR spectrum for the reaction product of SeO_2 with ethanol (Et–OH) leading to Et–O–SeOOH.

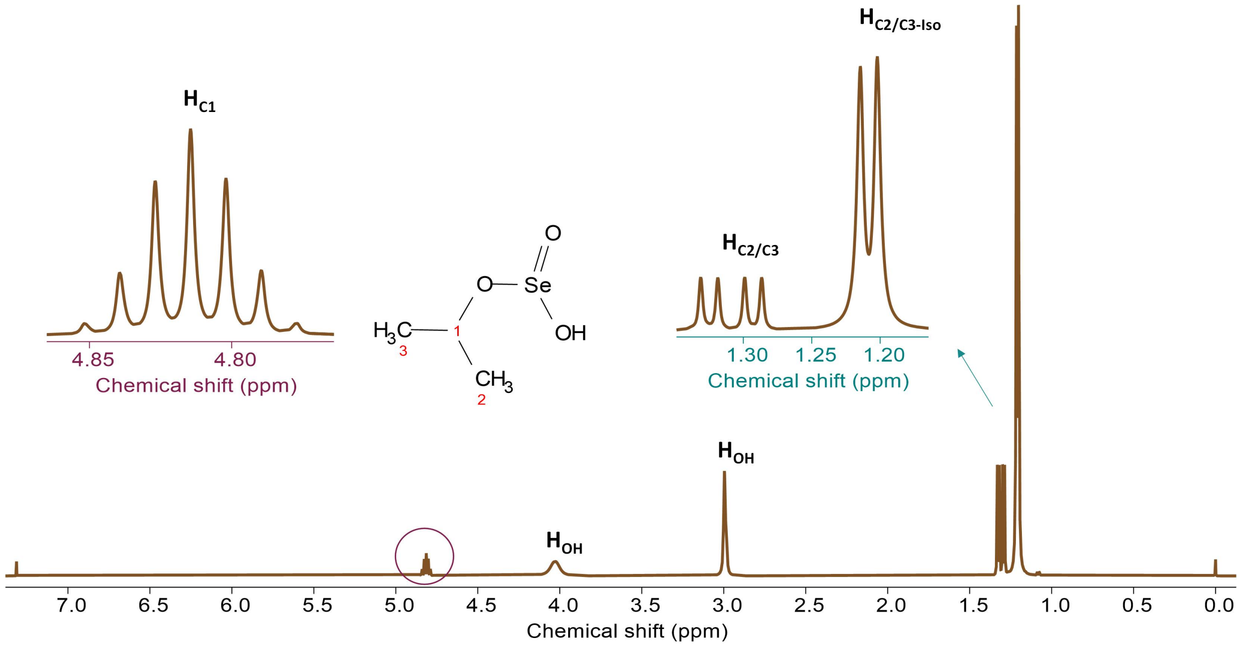

FIG. S6. ^{1}H NMR spectrum for the reaction product of SeO_2 with Isopropyl alcohol (Iso–OH) leading to Iso–O–SeOOH.

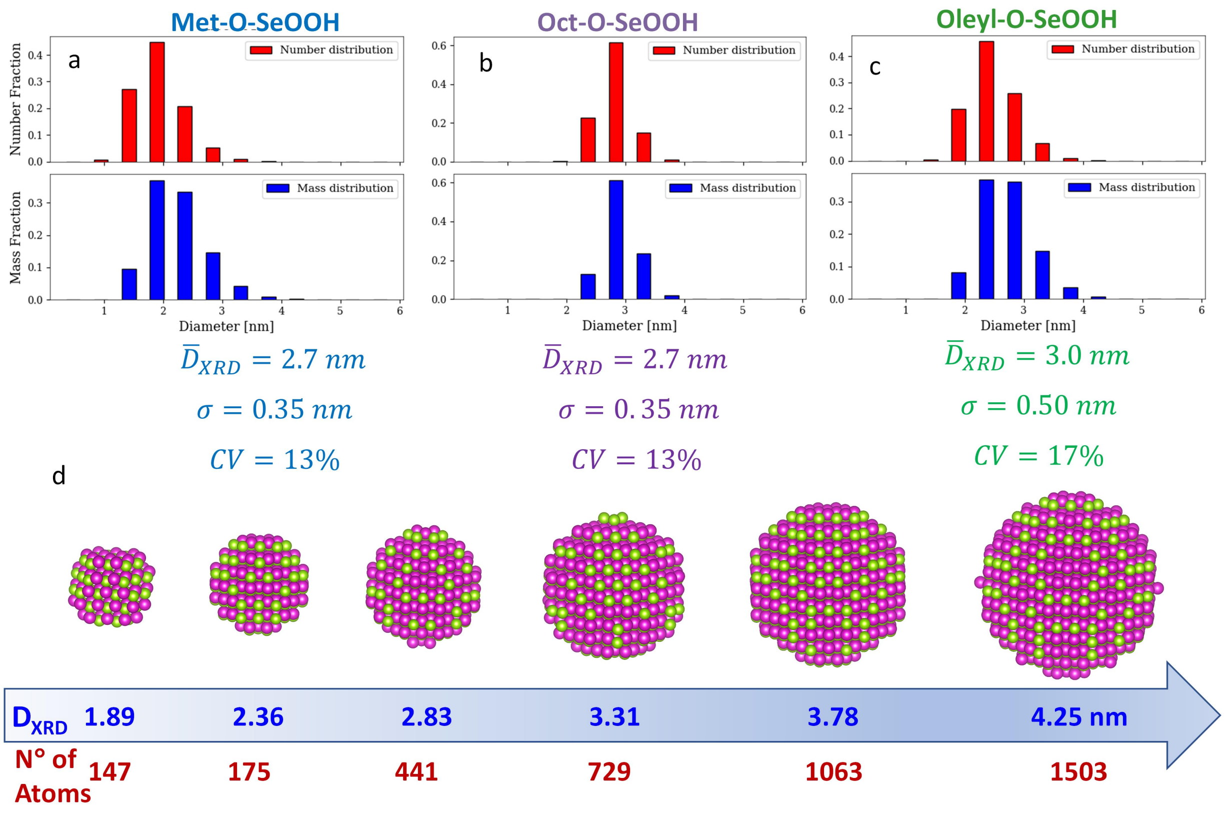

FIG. S7. Histogram of size distribution for samples Met–O–SOOH, Oct–O–SOOH, and Oleyl–O–SOOH showing the mean diameter size obtained from XRD ($\overline{D}_{XRD}$), standard deviation (σ), and coefficient of variation (CV). The Debussy 2.0,[1] software was used to fit the XRD patterns using Debye equation and a size distribution XRD pattern contribution based on the closed-shell nanoparticles with sizes and number of atoms displayed in the scheme below.

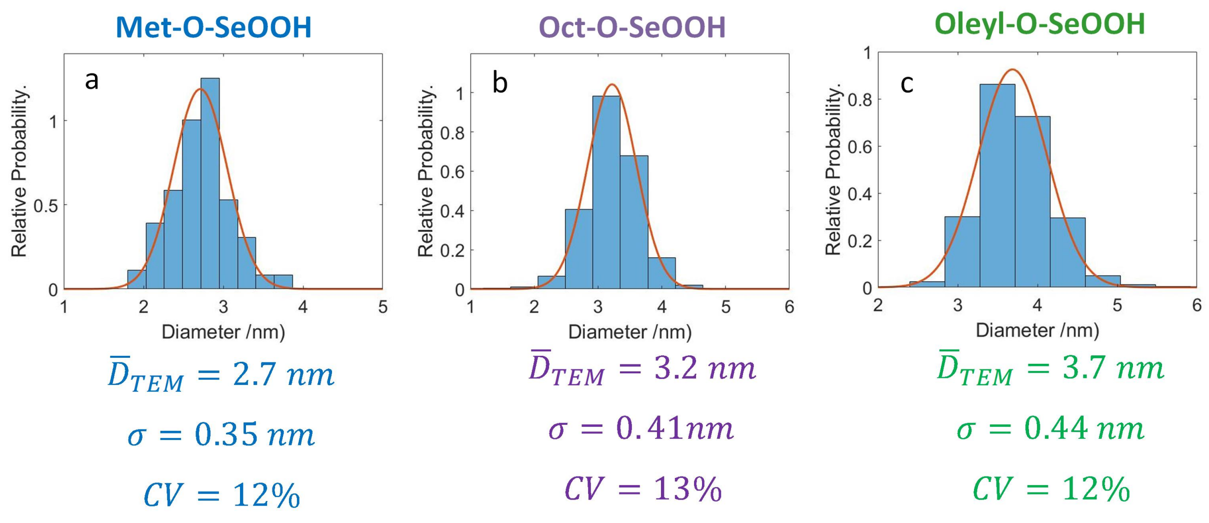

FIG. S8. Histogram of size distribution for samples Met–O–SOOH, Oct–O–SOOH, and Oleyl–O–SOOH showing the mean diameter size ($\overline{D}_{TEM}$), standard deviation (σ), and coefficient of variation (CV).

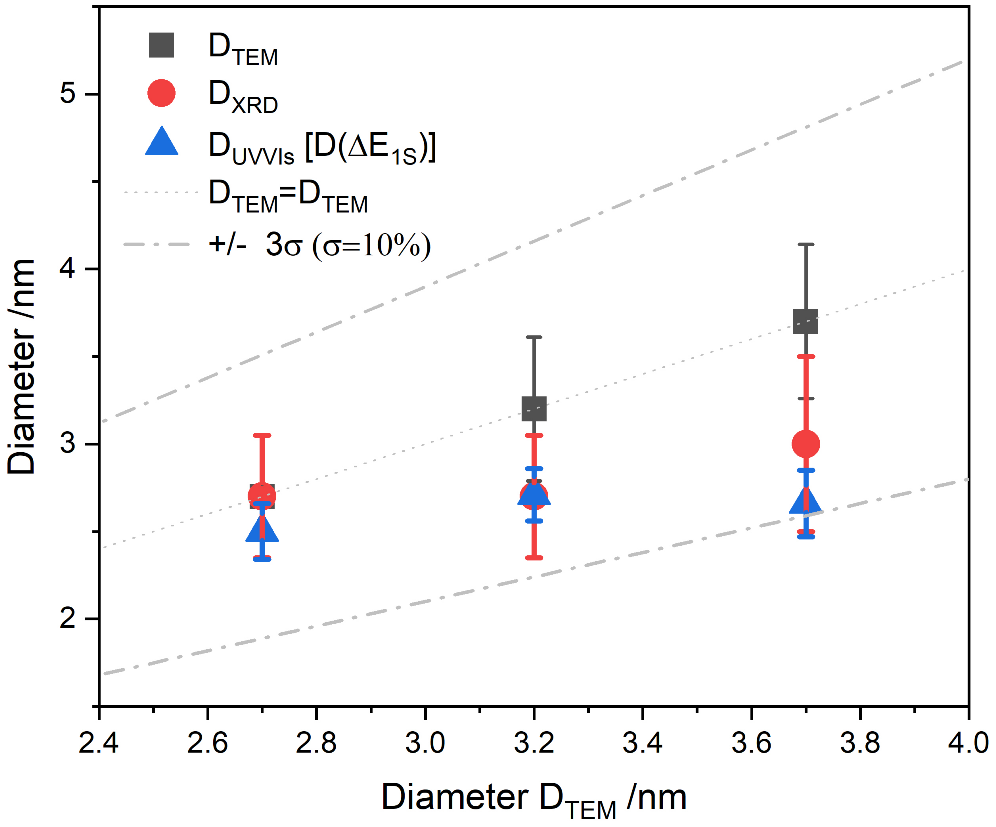

FIG. S9. CdSe QDs size determination for Met–O–SOOH, Oct–O–SOOH, and Oleyl–O–SOOH samples using Transmission Electron Microscopy images (TEM), X-ray Diffraction (XRD) via Debussy 2.0 software,[1] and UV-Vis absorption via Jasieniak and coworkers method.[2]

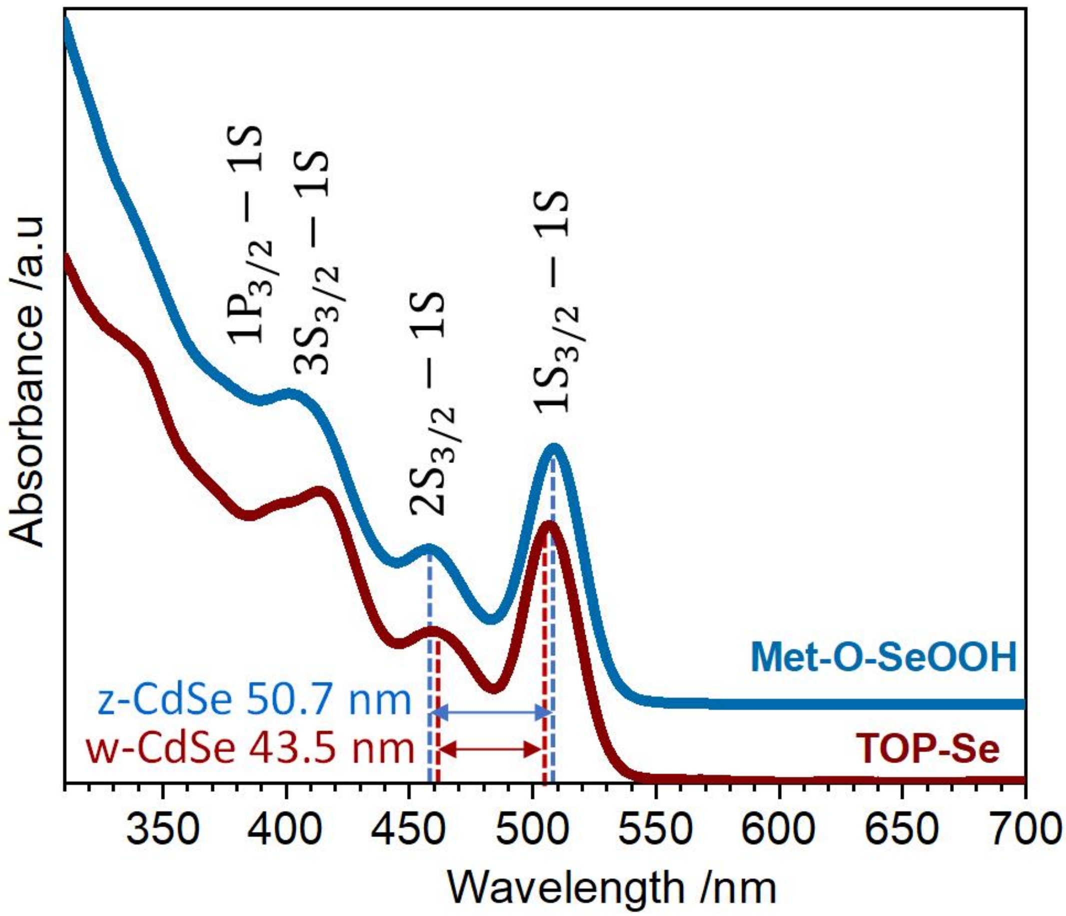

FIG. S10. UV-Vis spectra for CdSe QDs synthesised using standard TOP/TOPO method leading to w-CdSe structure (wurtzite, $P6_3mc$) and synthesized by alkyl selenite Met-O-SeOOH leading to z-CdSe Structure (zinc blende, $F\overline{4}3$m). As reported in the literature, the distance between the two excitonic absorption peaks $1S_e - 1S_h$ and $1S_e - 2S_h$ is higher for z-CdSe,[3] as observed for the CdSe QD synthesized in this work. Also, the size distribution of both samples are comparable, indicating that the R–O–SeOOH precursor leads to monodisperse nanoparticles.

TABLE S1. UV-Vis and Photoluminescence results for CdSe QDs synthesized with several Alkyl Acid Selenites precursos.

Sample	γ_{1st} (nm)	FWHM (eV)	PLQY (%)
Met–O–SeOOH	518.5	29.1	2.0
Et–O–SeOOH	539.6	31.7	1.6
Iso–O–SeOOH	556.5	32.1	2.0
TEG–O–SeOOH	572.1	49.0	1.0
Oct–O–SeOOH	528.6	33.2	5.0
Oleyl–O–SeOOH	536.7	45.0	15.0

TABLE S2. XPS parameters extracted from the deconvolution fitting of high-resolution Cd 3*d* and Se 3*d* peaks (Figure 7) showing the contribution of core and surface elements along with the Cd/Se atomic ratio for Met-O-SeOOH, Oct-O-SeOOH, and Oleyl-O-SeOOH samples.

Atom	BE (eV)	FWHM (eV)	Contribution (%)
Met-O-SeOOH Core			
Cd $3d_{5/2}$	405.33	0.87	46.6
Cd $3d_{3/2}$	412.09	0.88	
Se $3d_{5/2}$	53.98	0.94	54.3
Se $3d_{3/2}$	54.87	0.94	
Met-O-SeOOH Surface			
Cd $3d_{5/2}$	404.90	0.88	53.4
Cd $3d_{3/2}$	411.67	0.88	
Se $3d_{5/2}$	53.61	0.93	45.7
Se $3d_{3/2}$	54.43	0.93	
Oct-O-SeOOH Core			
Cd $3d_{5/2}$	405.22	0.88	43.6
Cd $3d_{3/2}$	411.98	0.88	
Se $3d_{5/2}$	53.89	0.92	53.5
Se $3d_{3/2}$	54.73	0.92	
Oct-O-SeOOH Surface			
Cd $3d_{5/2}$	404.76	0.88	56.4
Cd $3d_{3/2}$	411.53	0.88	
Se $3d_{5/2}$	53.44	0.91	46.5
Se $3d_{3/2}$	54.34	0.91	
Oleyl-O-SeOOH Core			
Cd $3d_{5/2}$	405.49	0.88	44.8
Cd $3d_{3/2}$	412.26	0.88	
Se $3d_{5/2}$	54.23	0.93	59.8
Se $3d_{3/2}$	55.10	0.93	
Oleyl-O-SeOOH Surface			
Cd $3d_{5/2}$	404.97	0.88	55.2
Cd $3d_{3/2}$	411.74	0.88	
Se $3d_{5/2}$	53.74	0.88	40.2
Se $3d_{3/2}$	54.60	0.89	

Sample	Cd/Se Ratio (% Atomic)
Met-O-SeOOH	60.0/40.0
Oct-O-SeOOH	58.8/41.2
Oleyl-O-SeOOH	63.9/36.1

REFERENCES

[1] A. Cervellino, R. Frison, F. Bertolotti, and A. Guagliardi, *DEBUSSY 2.0*: the new release of a Debye user system for nanocrystalline and/or disordered materials, Journal of Applied Crystallography **48**, 2026 (2015).

[2] J. Jasieniak, L. Smith, J. Van Embden, P. Mulvaney, and M. Califano, Re-examination of the size-dependent absorption properties of CdSe quantum dots, Journal of Physical Chemistry C **113**, 19468 (2009).

[3] F. Wu, Z. Zhang, Z. Zhu, M. Li, W. Lu, M. Chen, E. Xu, L. Wang, and Y. Jiang, Fine-tuning the crystal structure of cdse quantum dots by varying the dynamic characteristics of primary alkylamine ligands, CrystEngComm **20**, 4492 (2018).
